# A Keynesian perspective on the health economics of kidney transplantation would strengthen the value of the whole organ donation and transplantation service

**DOI:** 10.3389/fpubh.2023.1120210

**Published:** 2023-03-27

**Authors:** Francesca Leonardis, Lara Gitto, Evaldo Favi, Angelo Oliva, Roberta Angelico, Annapaola Mitterhofer, Irene Cacciola, Domenico Santoro, Tommaso Maria Manzia, Giuseppe Tisone, Roberto Cacciola

**Affiliations:** ^1^UTV Intensive Care Unit, Department of Surgical Sciences, Tor Vergata University, Rome, Italy; ^2^Dipartimento di Economia, Università degli Studi di Messina, Messina, Italy; ^3^General Surgery and Kidney Transplantation, Fondazione IRCCS Ca’ Granda Ospedale Maggiore Policlinico, Milan, Italy; ^4^Department of Clinical Sciences and Community Health, Università degli Studi di Milano, Milan, Italy; ^5^Coordinamento Trapianti, Policlinico Tor Vergata, Rome, Italy; ^6^Department of Surgical Sciences, HPB and Transplant Unit, University of Rome Tor Vergata, Rome, Italy; ^7^Nephrology and Dialysis Unit, University of Rome Tor Vergata, Rome, Italy; ^8^Department of Clinical and Experimental Medicine, Medicine and Hepatology Unit, University Hospital of Messina, Messina, Italy; ^9^Dipartimento di Medicina Clinica e Sperimentale, UOC di Nefrologia e Dialisi, Università degli Studi di Messina, Messina, Italy

**Keywords:** organ donation, organ transplantation, Keynesian model, public health, health economics, funding, savings, kidney transplantation

## Abstract

**Background:**

In this study, the Keynesian principle “savings may be used as investments in resources” is applied to Kidney Transplantation (KT), contextualizing the whole Organs Donation and Transplantation (ODT) service as a unique healthcare entity. Our aim was to define the financial resources that may be acquired in the form of savings from the KT activity.

**Methods:**

We analyzed registry and funding data for ODT in our region, between 2015 and 2019. Our hypotheses aimed to evaluate whether the savings would offset the Organ Donation (OD) costs, define the scope for growth, and estimate what savings could be generated by higher KT activity. To facilitate the evaluation of the resources produced by KT, we defined a coefficient generated from the combination of clinical outcomes, activity, and costs.

**Results:**

The ODT activity reached a peak in 2017, declining through 2018–2019. The savings matured in 2019 from the KT activity exceeded €15 million while the OD costs were less than €9 million. The regional KT activity was superior to the national average but inferior to international benchmarks. The estimated higher KT activity would produce savings between €16 and 20 million.

**Conclusion:**

The financial resources produced by KT contribute to defining a comprehensive perspective of ODT finance. The optimization of the funding process may lead to the financial self-sufficiency of the ODT service. The reproducible coefficient allows a reliable estimate of savings, subsequently enabling adequate investments and budgeting. Applying such a perspective jointly with reliable estimates would establish the basis for an in-hospital fee-for-value funding methodology for ODT.

## 1. Introduction

Organ Donation and Transplantation (ODT) services cover a critical and complex role in healthcare. A wealth of evidence over the years has defined with clarity the overall benefits for patients and the cost-effectiveness of Solid Organ Transplantation (SOT). Among all types of SOTs, kidney transplantation (KT) is recognized as being the best treatment for eligible patients with End-Stage Renal Disease (ESRD), but also, it achieves significant cost benefits in the form of savings when compared to the other types of renal replacement therapy (RRT). Different from ESRD, the actual financial implications of the management of patients with other organ failures, who would benefit from SOT, are not as similarly or reliably measurable.

The health economics of the three components of the ODT service, Organ Donation (OD), Organ Retrieval (OR), and SOT, are rarely contextualized as a whole, unique, and interdependent healthcare entity as most studies focus on the cost-effectiveness of specific organ transplants. Relevantly, the actual costs of OD and OR services are only occasionally included in the analysis. This is despite such costs are functional to the volume activity and success of SOT services.

The Coronavirus Disease 2019 (COVID-19) pandemic has mercilessly revealed several weaknesses in the healthcare systems. In particular, ODT services may be vulnerable, because of the organizational and funding complexity. In the context of current economic insecurity, with foreseeable financial repercussions, the Keynesian principle indicating that “savings may be used as investments in resources” would be applicable to healthcare in general, but more specifically to ODT. The application of such a principle is also sustained by post-Keynesian theories highlighting the relevance of health economics as belonging to the macroeconomic instead of the microeconomic sphere (1).

The present study aims to define the financial resources that may be acquired in the form of savings from the KT activity. The retrospective analysis of the whole ODT activity and costs in the Lazio region of Italy, between 2015 and 2019, also included the definition of a coefficient that allows to reliably estimate the potential resources that KT services may produce. In our hypotheses, we evaluate whether the savings generated by KT may offset the annual expenses for the OD services in our region. In order to define the scope for growth of KT activity, we compared the national and international benchmarks of ODT activity. Subsequently, we produced four different simulations of incremental KT activity that, following the application of the coefficient that we defined, have allowed us to estimate the hypothetical resources obtained. The suggested health economics perspective adds further strength to the established clinical value of ODT. In addition, it may facilitate the construction of a value-based model for the funding of the whole ODT. Such a perspective, potentially reproducible in any regional or national healthcare system, would be of critical relevance and pertinence in both developing and developed economies, as well as ODT programs.

## 2. Materials and methods

### 2.1. Description of the ODT services and funding in the Lazio region

In Italy, healthcare services are commissioned by the regional governments and authorities. The regional legislation is very similar across the 20 Italian regions. Although some variations may be observed, healthcare providers (HP) are funded by the Regional Commissioners of the Services (RCS) following regional legislation.

The Lazio region counts almost five million inhabitants which makes it like European countries such as Ireland, Finland, Norway, and Scotland. The five transplant centers of the Lazio region, one of which is exclusively dedicated to pediatric patients, are all located in the regional and national capital of Italy, Rome. The SOT activity is funded with a tariff system, therefore dependent on the volume activity of each transplant center. Each type of SOT is funded with a different tariff (kidney, €33,162; liver, €62,647; heart, €62,601; and lung, €72,572) (2). All Local Health Agencies (ASL) and their hospitals (HP), part of the National Health System (Sistema Sanitario Nazionale, SSN), receive funding for the OD from the RCS. There are two different types of payments. The first type of payment is allocated for the coordination of OD services in the form of a block payment. This payment is aimed to cover the costs of the providers for the personnel and the maintenance of the OD services. The second type, again in the form of a block payment, aims to reimburse the costs of the donation activity. The organs procured from any deceased-donor in Italy are allocated following established procedures agreed upon by the national and regional authorities. Therefore, any organ retrieved in any hospital of the SSN may be allocated in the same region, or to a patient in the transplant waiting list (TWL) of a different region. The management of the regional TWL, the allocation of organs, and the overall coordination of the OD and OR activities are under the responsibility of the National Centre for Transplantation (Centro Nazionale Trapianti, CNT) and the Regional Transplant Centre of Lazio (Centro Regionale Trapianti Lazio, CRTL) that is funded separately. The funding for the only regional histocompatibility laboratory (HL) is also separated. The costs related to the CRTL and the HL were €2.5 million and €280,000 per year, respectively (3). These costs were included in our analysis despite not being exclusive to SOT, as both absorb other activities concerning the donation and transplantation of bone marrow and tissues. The regional TWL for a SOT from a deceased-donor in Italy may include patients who are residents of any Italian region, as transplant centers offering KT, liver transplantation (LT), heart transplantation (HT), or lung transplantation (LuT) may not be available in the same region of residence or because of patient preference. The OR activity is not specifically financed as it is considered included in the organ transplantation tariff. Living donation does not appear to be included in the expenditures of the RCS. The service funding typology is as follows: OD coordination, block payment; OD activity, block payment; OR, not specifically funded; and SOT, tariff payment.

### 2.2. Data source

The ODT activity data were obtained from the CRTL database and cross-referenced for accuracy with the annual reports of the CNT (4). The data relating to the funding and finance of the ODT service were obtained from the financial regional legislation published by the RCS in the Official Gazette (2, 5, 6).

### 2.3. Activity analysis of ODT

We analyzed the volume activity of the ODT services in the Lazio region of Italy for 5 consecutive years (namely, from 1 January 2015 to 31 December 2019). The years 2020 and 2021 have not been included because the COVID-19 pandemic did substantially influence ODT activity globally (7–9). The OD activity was evaluated through the number of utilized donors (UD) defined as donors from whom at least one organ was transplanted (10). Our analysis of SOT included the number of all organs transplanted in the regional transplant centers.

### 2.4. Cost analysis of ODT

The cost analysis was based on the officially documented expenditures for the ODT services reported in the regional legislation (2–6). The OD costs were divided into two components: activity and coordination. The SOT cost was produced by applying the regional tariff to all organs transplanted in the study period. The savings produced by KT were calculated from two established pieces of evidence. The first one is represented by the minimal savings obtained per year per functioning kidney transplant (FKT), and after the first year of transplant, is indicated €25,000 (11–14). The second one is represented by the minimum predictable efficacy of treatment (EoT) based on a minimum graft survival (GS) of 80% every year for the first consecutive 5 years (15, 16). Such established evidence allowed the definition of the Kidney Transplant Coefficient of Value (KTCoV) that was also used in our analysis.

### 2.5. Parameters and formulas

Estimated Functioning Kidney Transplant (eFKT) = 80% of total number of KT = (1,156/100) × 80 = 924.8;

Estimated Non-Functioning Kidney Transplant (eNFKT) = 20% of total number of KT = (1,156/100) × 20 = 231.2;

The estimated Gross Savings (eGrSav) was calculated from the difference between the savings produced by the eFKT and the cost of the eNFKT: (eFKT × 25,000) − (eNFKT × Tariff) = eGrSav = (924.8 × 25,000) − (231.2 × 33.162) = 23,120,000–7,667,054.4 = €15,452,945.6;

The Estimated Net Savings (eNSav) were obtained from the balance between eGrSav and the costs of OD, CRTL, and HL: eGrSav − (OD + CRTL + HL) = eNSav (€) = 15,452,945.6 − (5,832,590 + 2,500,000 + 280,000) = €6,840,355.6;

The KTCoV was determined by dividing the eGrSav by the total number of KT performed: eGrSav/number of KT = KTCoV = 15,452,945.6/1156 = €13367.6.

### 2.6. Hypotheses

*H1*: Would the savings matured in 2019, through the KT activity of 2015–2019, offset the OD costs for 2019?

We evaluated whether the eGrSav and eNSav produced by the number of KT in the study period with minimal expected EoT may offset the annual costs of the OD services.

*H2*: How does the Lazio region ODT activity compare nationally and internationally?

In order to define the scope for the growth of KT activity, we compared the type and rate/per million population (pmp) of UD and SOT observed in the Lazio region in 2019 (the last year of the study) with the Italian national average and other European countries comparable to Italy for the number of inhabitants and ODT activity. We used the data produced yearly by the International Registry of Organ Donation and Transplantation (IRODaT) (17).

*H3*: What savings would generate a higher KT level of activity (LoA) in the Lazio region?

We produced four different simulations with an incremental number of KT over a 5-year period. Subsequently, we applied the KTCoV to the hypothetical number of KT.

## 3. Results

### 3.1. OD activity

The data analyzed from the official sources revealed that in the study period, the number of UD increased till 2017; thereafter, the following year remained identical, but with an inferior number of organs utilized. In 2019, both the number of UD and organs transplanted decreased. The average rate of UD/pmp ranged between 19.1 and 24.4. The average number of organs utilized per UD was constant in the 5 years, and it was 2.8 per UD. As shown in Table 1, kidneys were the most utilized among all organs retrieved in the study period (917/1590; 57.6%).

**Table 1:** Activity and costs of Organ Donation and Transplantation service in Lazio region, Italy, between 2015 and 2019.

	2015	2016	2017	2018	2019	2015–2019
UD (n)	98	117	122	122	105	561
UD/pmp (n)	19.6	23.4	24.4	24.4	21	22.6 ± 2.2
KT from UD (n)	160	189	208	197	163	917
LT from UD (n)	86	99	99	90	87	461
HT from UD (n)	19	33	27	12	17	108
LuT from UD (n)	13	24	22	22	23	104
SOTs from UD (n)	278	345	356	321	290	1,590
SOTs per UD (n)	2.8	2.9	2.9	2.6	2.9	2.8 ± 0.1
Funding of activity (€)	2,820,590	2,820,590	2,820,590	2,820,590	2,820,590	14,102,950
Funding of coordination (€)	3,012,000	3,012,000	3,012,000	3,012,000	3,012,000	15,060,000
Total cost of OD (€)	5,832,590	5,832,590	5,832,590	5,832,590	5,832,590	29,162,950
Average cost per UD (€)	59,517	49,852	47,809	47,809	55,548	51,984
KT (n)	164	241	262	266	223	1,156
KT tariff cost (€)	5,438,568	7,992,042	8,688,444	8,821,092	7,395,126	38,335,272
LT (n)	141	163	145	149	155	753
LT tariff cost (€)	8,833,227	10,211,461	9,038,815	9,334,403	9,710,285	47,128,191
HT (n)	28	30	26	17	18	119
HT tariff cost (€)	1,754,116	1,878,030	1,627,626	1,064,217	1,126,818	7,450,807
LuT (n)	12	16	10	8	12	58
LuT tariff cost (€)	870,864	1,161,152	725,720	580,576	870,864	4,209,176
Total SOTs (n)	345	450	443	440	408	2086
Total SOTs tariff cost (€)	16,896,775	21,242,685	20,080,605	19,800,288	19,103,093	97,123,446

UD, utilized donor; n, number; pmp, per million population; KT, kidney transplant; LT, liver transplant; HT, heart transplant; LuT, lung transplant; SOT, solid organ transplant; OD, organ donation.

### 3.2. SOT activity

The number and type of transplants performed varied across the years. All deceased-donors were from donation after brain death (DBD) and the overall rate of KT from living-donor (LDKT) was 15.2% (176/1156). The number of LT was substantially superior to the number of UD and procured livers in the region. The activity of cardiothoracic transplantation shows that the HT activity, after peaking in 2016, progressively reduced by 40% in 2019, while the number of LuT in 2019 was the same as in 2015 (Table 1).

### 3.3. Cost analysis

The OD services received a fixed payment of €5.8 million per year, for financing both coordination and activity. The SOT is financed *via* tariff payments. In our series, it ranged between €16.9 million and €19.1 million, reaching a peak in 2016 of €21.2 million. The cumulative tariff cost of the whole SOT activity in the study period was calculated at €97.1 million. The annual average was €19.4 million.

### 3.4. Hypothesis 1

The eGrSav calculated from the savings produced by the eFKT and the costs of the eNFKT was €15.5 million. The documented costs per year related to OD services including CRTL and HL were in total €8.6 million. Our calculations indicate that the eNSav in 2019 matured after 5 years of KT activity was €6.8 million (Table 2).

**Table 2:** Calculation of estimated gross savings and estimated net savings obtained in 2019.

	Savings	Costs	Balance
Estimated functioning kidney transplant (n) × Annual saving (€) (924.8 × 25,000)	23,120,000	–	–
Estimated non-functioning kidney transplant (n) × Tariff (€) (231.2 × 33,162)	–	7,667,054	–
Estimated gross saving (€)	–	–	15,452,946
Kidney transplant coefficient of value* (€)	13367.6	–	–
Cost of organ donation in 2019 (€)	–	5,832,590	–
Cost of CRTL (€)	–	2,500,000	–
Cost of histocompatibility laboratory (€)	–	280,000	–
Cumulative cost of organ donation (€)	–	–	8,612,590
Estimated net saving (€)	–	–	6,840,356

* KTCoV: Estimated Gross Saving/Total number of kidney transplants = 154,529,456/1156.

KTCoV, kidney transplant coefficient of value; OD, organ donation; CRTL, Centro Regionale Trapianti Lazio.

### 3.5. Hypothesis 2

The analysis of the OD activity as reported by IRODaT includes the rates of donation after circulatory death (DCD) and UD after DCD. The KT activity was divided between KT from deceased-donor (DDKT) and LDKT. All other SOT rates/pmp are also included.

According to the database of IRODaT, the European ODT programs with the highest LoA and with a comparable population to Italy are the United Kingdom (UK), France, and Spain. The ODT activity observed in the Lazio region and the comparison with the national averages of Italy, the UK, France, and Spain is summarized in Table 3. The overall rate of UD in Lazio was 22.5/pmp which is slightly inferior to the national average (23.2/pmp), and very close to the lower value of the international comparison range (22.8–42.8/pmp). It is noticeable that there were no DCD in Lazio. The KT rate in Lazio is 46.2/pmp, which is superior to the national average (36.1/pmp), but inferior to the lower value of the international comparison range (54.4–73.7/pmp). The high rate of LT (31/pmp) is noticeable in Lazio compared to national (22/pmp) and international (14.5–26.4/pmp) rates. In order to achieve a KT rate of 54/pmp, and be in the range of the high-performing European KT programs, it will be needed to perform 270 KT/year. Hence, it will require an increase of 39 more KT per year (16.8% increase).

**Table 3:** Summary of 2019 Organ Donation and Transplantation activity (rate/pmp) in Lazio Region, Italy, UK, France, and Spain.

	Lazio	Italy	UK	France	Spain
Total UD/pmp	22.5	23.2	22.8	28.7	42.8
UD DCD type (n)	0	1.08	9.07	2.89	13.08
Total kidney transplant/pmp	46.2	36.1	54.4	55.6	73.7
Deceased-donor kidney transplant (n)	38	30.4	39.2	47.8	66.5
Living-donor kidney transplant (n)	8.2	5.7	15.2	7.8	7.2
Liver transplant (n)	31	22	14.5	20.7	26.4
Heart transplant (n)	3.6	4.14	2.8	6.63	6.47
Lung transplant (n)	2.4	2.58	2.5	6	9.03

ODT, organ donation and transplantation; UD, utilized donor; pmp, per million population; DCD, donation after circulatory death.

### 3.6. Hypothesis 3

The four simulations ranged from an average of 240 to 300 KT per year that, if maintained for 5 consecutive years, would produce between 1,200 and 1,500 KT. The calculation of the hypothetical eGrSav produced by the increased activity, achieved by multiplying the hypothetical number of KT in 5 years by the KTCoV (€13367.6), indicates that the hypothetical eGrSaV ranges between €16.1 million and €20.1 million, while the eNSav ranges between €7.4 million and €11.5 million per year (Table 4).

**Table 4:** Simulations of increased kidney transplantation activity with estimated gross and net savings.

	Actual (2015–2019)	Simulation 1	Simulation 2	Simulation 3	Simulation 4
	KT 46.6/pmp	KT 48/pmp	KT 52/pmp	KT 56/pmp	KT 60/pmp
Average annual number of KT required (n)	231	240	260	280	300
Annual increase rate required (%)	–	3.9%	12.5%	21.2%	29.8%
Number of KT in five years (n)	1,156	1,200	1,300	1,400	1,500
Estimated Gross Saving* (€)	15,452,946	16,051,920	17,389,580	18,714,640	20,064,900
Organ Donation Cost (€)	5,832,590	5,832,590	5,832,590	5,832,590	5,832,590
CRTL and Histocompatibility Laboratory (€)	2,780,000	2,780,000	2,780,000	2,780,000	2,780,000
Estimated Net Saving (€)	6,840,356	7,439,330	8,776,990	10,102,050	11,452,310

* Estimated gross saving: (number of kidney transplant) × (KTCoV).

KT, kidney transplant; pmp, per million population; KT, kidney transplant; CRTL, Centro Regionale Trapianti Lazio; KTCoV, kidney transplant coefficient of value (€13376.6).

## 4. Discussion

### 4.1. The rationale for a Keynesian perspective

“He must study the present in the light of the past for the purposes of the future” John M. Keynes.

The expenditures in financing the regional ODT service are clearly reported. However, some critical aspects of the actual costs of the service incurred by HP may not be fully reflected in the current combination of block and tariff payments. This represents a limitation of our study, as much as a burden in service costing and budgeting.

The current finance model of the care pathway, starting from the identification of potential donors to the ultimate number of UD, is based on block payment. This is despite a critical amount of expenses encountered by HP are dependent on the volume of activity produced. The most critical costs related to OR activity are included in the SOT tariffs. Such costs may translate into highly onerous commitments for HP. Undoubtedly, the financial implication of OR activity would benefit from a broader national strategy, rather than a regional or HP-based organization. In addition, the balance of organs exchanged between regions, with the payment of tariffs for patients transplanted outside the regional services, may be linked to relevant, but not fully accounted for, financial aspects of ODT services.

In our analysis, we have intentionally under-represented the EoT of KT basing our calculations on a GS rate of 80% from the first year after KT. We aimed to define the minimum savings that would be acceptable as a reliable estimate. Numerous scientific and governance reports confirm that stratified GS rates are substantially superior; therefore, suggesting that the actual savings produced by successful KT may be higher than that indicated by our analysis (15, 16). In addition, the savings produced by successful KT functioning for more than 5 years (in particular, those from standard criteria DBD and living-donors) have not been intentionally stratified in our analysis. The cost-effectiveness of other SOTs demonstrated by the quality-adjusted life years (QALY) is not included because the actual savings produced are not as clearly measurable as for KT. However, growing evidence indicates that LT may produce robust savings after the initial costs linked to the surgical procedure are compensated. Furthermore, the costs related to the management of patients with liver failure who would benefit from LT, as much as the costs of death caused by liver failure complications, are substantial (17–19).

The fee-for-service remains the most used method of financing healthcare services in Italy and internationally. However, the application of such a method for financing ODT services may not fully reflect the current requirements and the future challenges that the whole service will be confronting in a global healthcare crisis such as the one we are already witnessing (20, 21). Consequently, the interdependence of the three components of ODT (OD, OR, and SOT) that extends beyond its scientific and clinical boundaries, reaching organizational aspects of the service, may be optimized with a more comprehensive and integrated perspective of the financial processes of the whole ODT. Addressing the savings produced by KT as resources for ODT components, such as OD and OR, is fully justified by the fact that KT is the major beneficiary of both services. This is also indicated in our series, where on average five UD produced eight KT, four LT, one HT, and one LuT. In addition, the demonstrable savings obtained by a globally reproducible EoT offer a unique opportunity in healthcare in defining the actual value of a multidisciplinary service through the contextualization and merging of clinical benefits and finance.

Our analysis indicates that the fixed funding for OD, allocated to regional HP through block payments, was not associated with the progressive growth of UD as observed elsewhere (22). Although the actual recession of UD might not be exclusively ascribed to unchanged funding, this observation alone may prompt the evaluation of adequate resources and workforce.

The rate of 46.2 KT/pmp observed in the Lazio region is not associated with a similarly high rate of UD. Such apparent inconsistency may be explained by an increased allocation of kidneys to satisfy the increasing demand of a large regional TWL of 900 patients at present.

According to our analysis, it appears that the estimated savings produced by KT alone in 2019 may comfortably offset the current OD services expenditures, including CRTL and HL costs, providing also almost €7 million as a resource for wider healthcare.

### 4.2. Hidden costs and hidden savings

“It is better to be roughly right than precisely wrong” John M. Keynes.

The expenditures of RCS in financing the regional ODT service are clearly reported. However, some critical aspects of the actual costs of the service incurred by HP may not be fully reflected in the current combination of block and tariff payments. This represents certainly a limitation of our study, as much as a burden in service costing and budgeting.

The current finance model of the care pathway, starting from the identification of potential donors to the ultimate number of UD, is based on block payment to HP. This is despite a critical amount of expenses encountered by HP are dependent on the volume of activity produced. Arguably, the high variability of the costs may not be fully honored only with block payments, particularly so, to HP with the high-volume activity of OD. Conversely, HP with low-volume activity receives a block payment that may exceed the costs encountered. The most critical costs related to the OR activity are included in the SOT tariffs. Such costs may translate into highly onerous commitments for individual HP, even if benefitting from SOT tariffs. Undoubtedly, the financial implication of OR activity would benefit from a broader national rather than regional or HP-based strategy and, as importantly, commissioning for cost optimization. In the context of regionalized healthcare, the balance of organs exchanged between regions, as well as the payment of tariffs for patients transplanted outside the regional services, may be linked to a relevant, but not fully accounted, financial aspect of the ODT services.

In our analysis, we have intentionally under-represented the EoT of KT basing our calculations on a GS rate of 80% from the first year after KT. We aimed to facilitate the calculations for defining the minimum savings that would be accepted as a reliable estimate. Numerous scientific and governance reports identify stratified GS rates as substantially superior; therefore, suggesting that the actual savings produced by successful KT may be higher than that indicated by our analysis (15, 16). In addition, the savings produced by successful KT functioning for more than 5 years (in particular, those from standard criteria DBD and living-donors) have not been accounted for in our analysis. The cost-effectiveness of other SOTs demonstrated by the related quality-adjusted life years (QALY) is not included because the actual savings produced are not as clearly measurable as for KT. However, there is growing evidence indicating that LT may produce robust savings after the initial costs linked to the surgical procedure are compensated. Furthermore, the costs related to the management of patients with liver failure who would benefit from LT, as much as the costs of death caused by liver failure complications, are substantial (17–19).

Conciliating the actual costs encountered by HP with the funding for ODT and the savings produced by SOT would be crucial for future spending reviews. In Table 5, we highlighted aspects of the service not clearly captured by the current financing process.

**Table 5:** Costs and Savings related to Organ Donation and Transplantation not clearly reported.

Hidden costs	Hidden savings
Identification of potential donor:	Savings from Graft survival >80%
Multidisciplinary consultations	
Usage of ICU beds	
Evaluation of potential donor:	Savings from Graft survival >5 years
Laboratory	
Radiology	
Samples transportation	
Organ Retrieval:	Savings from LT/HT/LuT
Workforce	
Transportation of teams	
Transportation of organs	
Operative room	
Organs Imported from other regions	Organs exported to other regions
Tariff paid for patients resident in Lazio who were transplanted in other regions	Tariff received for patients resident in other regions who were transplanted in Lazio
Living Donation:	
Workforce	
Assessment	

ICU, intensive care unit; LT, liver transplant; HT, heart transplant; LuT, lung transplant.

### 4.3. Potential objectives

“The importance of money flows from it being a link between the present and the future” John M. Keynes.

The KTCoV facilitated the calculations of hypothetical savings produced in the study years and in the four simulations. Producing a reliable estimate of the minimum savings enables the definition of clear objectives of growth and related budget. The factors used for the definition of the KTCoV may vary in time, according to the ODT program, tariff, or EoT. In addition, the estimated savings may change in time due to inflation or discount rates. Similarly, the tariff for KT may change or vary between regional or national ODT programs. Furthermore, the application of specific GS rates produced by detailed governance reports allows an even more accurate estimate, as much as a more granular evaluation of the savings produced, according to different GS rates (23). Therefore, independently of the corrections that may be required, the adaptability of the formula determining the KTCoV suggests that it may be used by the commissioners and stakeholders of any regional or national ODT program.

The critical mass of patients waiting for a KT defines “*per se*” the scope for the growth of KT services as, if not transplanted, they will remain exposed to suspension or removal from the lists, or death while waiting (4, 24).

The realistic feasibility of obtaining higher LoA of KT may be identified when contextualizing the KT/pmp of the Lazio region with international rates; in particular, when comparing it with the UK. In fact, despite the UK suffering the lowest rate of UD from DBD (13.7/pmp), the actual rate of KT is remarkably higher than Lazio and the Italian national average. Such observation proves that LDKT and DCD may substantially contribute to reaching high rates of KT and may be potentially reproducible also in other ODT programs (25). A substantial contribution to the growth of KT may be offered by a living donation. Although it may be conceptually acceptable to rely on LDKT growth in order to achieve a desired objective, it may be difficult, albeit possible.

The allocation of resources in the context of a restrained healthcare budget represents a remarkable challenge; more relevantly so during the ongoing COVID-19 pandemic associated with global economic uncertainty. In our simulations, the robust, yet hypothetical, savings achieved through an incremental number of KT ranges between €7.4 million and €11.5 million per year; undoubtedly representing a precious resource.

**Figure 1:**
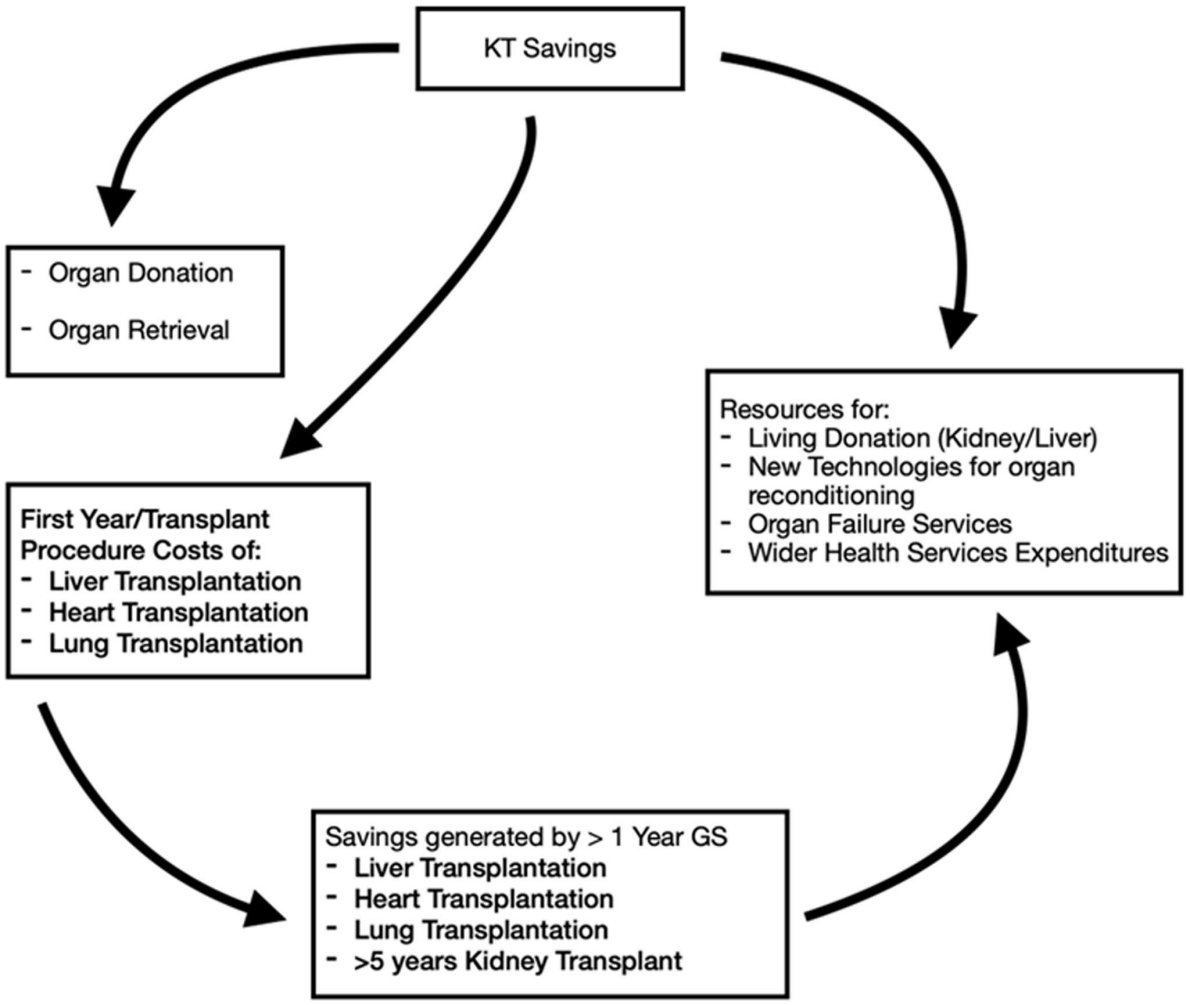
Potential flow of resources originating from the implementation of kidney transplant (KT) activity.

Applying the principle of considering the whole ODT as a unique healthcare entity that embraces interdependent services, we reproduce in Figure 1 a potential flow of resources.

### 4.4. A value-based approach for ODT

“The difficulty lies not so much in developing new ideas as in escaping from old ones” John M. Keynes.

Value-based care differs from a fee-for-service in which providers are paid based on the number of healthcare services they deliver. In value-based healthcare, the “value” is derived from measuring health outcomes against the cost of delivering the outcomes (26). Consequently, it may be sustained that ODT, because of its complexity and the highly successful practice of SOT, consisting of high rates of survival of both patients and grafts, would benefit from being funded as an in-hospital service with a comprehensive fee-for-value rather than a compartmentalized fee-for-service funding method.

Our study has reaffirmed the rather unique attributes of KT that embrace remarkably high success rates with the production of demonstrable financial resources. Conceivably, these resources could represent the “economic engine” for the whole of ODT. In this context, the perspective of addressing the health economics of the ODT services comprehensively, instead of a fragmented structure, would contribute to defining the overall economic benefits of ODT, as well as it would lay the fundament for a fee-for-value funding methodology for the whole ODT. The cost-effectiveness of SOT demonstrated through the QALY linked to the concept of “willingness-to-pay” may represent a true limit (27). In fact, such a concept inevitably will be confronted in the future with the actual “capacity-to-pay.”

The yearly eGrSav produced by KT beyond rendering OD financially independent could also contribute to offset OR activity, contributing partly or entirely to the procedure-related costs of other SOTs; therefore, further enhancing the “value” of the whole ODT that may be addressed as a financially self-sufficient healthcare entity.

The implementation of effective strategic growth is certainly possible also by expanding the same governance structure that ensured globally reproducible success rates toward those parts of the service ensuring access to transplantation, adequate infrastructures, and workforce.

Our study does not identify a new flow of money. Instead, it offers an instrument to reliably estimate the financial benefits produced by KT that, with adequate corrections, may be potentially applied to other Italian regions, nationally and internationally. In Italy, OD and SOT are included in those services recognized as essential that will cost at least €200 million per year (28, 29). Although ODT may represent a small proportion of such costs for the taxpayers, addressing ODT as a national resource and a financially self-sufficient service may realistically represent an enormous benefit for patients, wider ODT community, commissioners, and HP.

## Data availability statement

The raw data supporting the conclusions of this article will be made available by the authors, without undue reservation.

## Author contributions

FL, LG, IC, and RC: rationale of the study and original draft of the manuscript. EF, AM, DS, TM, GT, and RC: final revision. FL, LG, AO, RA, and IC: data collection and interpretation. RA, TM, and RC: literature review. EF: editing. EF, AM, and RC: supervision. AO, DS, and GT: logistics. All authors contributed to the article and approved the submitted version.

## Funding

Publication’s costs were funded by Grant Ricerca Corrente, Italian Ministry of Health.

## Conflict of interest

The authors declare that the research was conducted in the absence of any commercial or financial relationships that could be construed as a potential conflict of interest.

## Publisher’s note

All claims expressed in this article are solely those of the authors and do not necessarily represent those of their affiliated organizations, or those of the publisher, the editors and the reviewers. Any product that may be evaluated in this article, or claim that may be made by its manufacturer, is not guaranteed or endorsed by the publisher.
